# Analysis of Annual and Seasonal Precipitation Variation in the Qinba Mountain area, China

**DOI:** 10.1038/s41598-020-57743-y

**Published:** 2020-01-22

**Authors:** Yannan Zhang, Chuan Liang

**Affiliations:** College of Water Resource and Hydropower, Sichuan University, 610065 Chengdu, China

**Keywords:** Hydrology, Information technology

## Abstract

In this study, the spatial and temporal characteristics in precipitation extremes, total precipitation, and the seasonality of precipitation of the Qinba Mountain in China were investigated from 1961 to 2015, based on daily precipitation data of 37 meteorological stations. The results from our study suggest that: the annual precipitation in the area varies between 645 mm and 2175.2 mm, with the minimum annual precipitation occurred in 1997, and the maximum annual precipitation, in 1963. Between 1961 and 2015, a significant decreasing trend was observed in the annual precipitation, suggesting a decrease of −21.1 mm/a. The spatial distribution of precipitation in the Qinba Mountain area increased from the north to the south, showing an obvious difference in precipitation between the two areas. For extreme indices, the trends of annual total wet-day precipitation (PRCPTOT), number of heavy precipitation days (R10mm)and consecutive wet days (CWD)showed a downward trend, while the other extreme indices had an upward trend. The results from our research not only help the researchers to understand the characteristics of precipitation, but also provide crucial information for the policy makers to make better decisions, in future.

## Introduction

Global warming has made great changes in the regional atmospheric circulation and the water circulation, resulting in redistribution of precipitation around the world^1^. The latter has led to an increase in the number of natural disaster. During the last decades, the climate change has attracted widespread attention in the globalisation of society^2^. The precipitation pattern change is one of the main factors of the regional climate change, and can be also considered as a key factor in the economic development and the environmental change^3^. The change in precipitation pattern can cause catastrophic event and affect environment and society by increasing the probability of flooding in the area in time of heavy precipitation, or water deficiency as a result of the decrease in precipitation. Indeed, both floods and droughts can cause different levels of disasters. Therefore, it is important to study the effects of changes in precipitation characteristics on the ecology, environment, and economy of the area^4^.

China is a large population and agriculture is a vital industry in the country. Therefore, studying the changing trend of the precipitation is of great significance to the regional agricultural development and water resources allocation^5,6^. During the recent decades, the total annual precipitation has slightly changed, in China and the spatial distribution of precipitation is very different in different areas within the China^7^. In the southwest, an increase in precipitation was observed both in the summer and the winter^8^. However, in the eastern region, significant seasonal variations in precipitation are recorded^9^. In the Yellow River Basin, the annual precipitation decreased significantly, in contrast, a significant increase was observed in the Yangtze River Valley^10^.

Mountainous area is a remarkable feature of the Chinese topography, which accounts for 2/3 of the total area of the country. In the vast mountainous area with the inconvenient transportation, the development of regional agriculture lags behind^11^. At present, the economy of mountain areas mainly depends on the forest products, the mineral resources, the water power and the tourism resources. Therefore, the climate and rainfall change in the mountain areas have a direct impact on the economy of mountain areas^12^. The current literature reviews suggest that many studies have been carried out on the precipitation in mountain areas. Naresh Kumar Ashok *et al*. (2016) studied the temporal variation in the precipitation, considering the influence of the Western Himalayan Region, from 1857 to2004^13^; Kexin Zhang *et al*. (2014) explored the spatial distribution and the temporal trends of the extreme precipitation in the Hengduan Mountains region, from 1961 to 2012. They suggested regional differences in regards with the influence of topography in the Hengduan Mountains region^14^. Junfeng Liu *et al*. (2016) studied the precipitation in the Qilian Mountains^15^. The precipitation changes of the Altai Mountains was subjected to study by Malygina Natalia *et al*. and the influence of atmospheric circulations during 1959 to 2014 on the precipitation pattern was explored in depth^16^. Nina K Kononova *et al*. studied the effects of atmospheric circulation on the summertime precipitation variability, in Tianshan Mountains^17^.

Qinba Mountain area as a typical mountain area with limited access to the land is a national poverty area, which lies in a heavy rain zone. The area has a complex terrain, and suffers from floods, landslides, and other natural disasters such as mudslides^18^. Due to high intensity rainfall during a short period of time, slope mud is formed the stone flow and landslide, which cause serious disasters and losses^19^. It is now widely recognized that the extreme events are very likely to increase, as a result of the climate change. Such a catastrophic events have much greater impacts on the environment and the society, than a small shift in the mean values^20^.

The precipitation around the Qinba Mountain area has been redistributed under the influence of the global warming, during recent years^21,22^. Hence, the aim of this research is to study the temporal and spatial characteristics of the precipitation in the Qinba Mountain area, and to provide a reference for predicting the occurrence of extreme precipitation^23^.

The precipitation data from 37 stations were selected in Qinba Mountain and surrounding areas. The homogeneity of these data was tested and the data quality was significantly improved after correction. Subsequently, temporal changes in the annual and seasonal precipitation during 1961 to 2015, as well as the trend of eight extreme precipitation indices and distributions of the temporal change in precipitation were examined in this work. The outcome from this research can be served as a reference for the researchers in this area and provides sufficient information for the policy makers in fields of agriculture and water resource management.

## Results

### Temporal trends of precipitation change

Based on the daily precipitation data between 1961 and 2015, the monthly mean precipitation is calculated in order to study the monthly precipitation distribution. During 1961–2015, the annual mean precipitation in the study area was 1039 mm (Table 1). Among the four seasons, the precipitation during the summer (June- August), and the autumn (September- November) accounts for 35.1% and 24.2% of the annual precipitation, respectively. Therefore, the precipitation in the QM is mainly concentrated in the summer, and July has the most precipitation with the ratio of 14.64% of the annual precipitation, and February has the least precipitation with the ratio of 3.51%.

**Table 1:** The precipitation amount in each month in the study area, during 1961–2015.

Month	Precipitation (mm)	Ratio of Annual Precipitation (%)
January	42.6	4.1
February	36.5	3.51
March	56.5	5.44
April	78.7	7.57
May	106.3	10.23
June	106.4	10.23
July	152.1	14.64
August	124.2	11.95
September	122.1	11.75
October	87.8	8.45
November	71.4	6.87
December	54.4	5.24
Annual	1039	100

**Figure 1:**
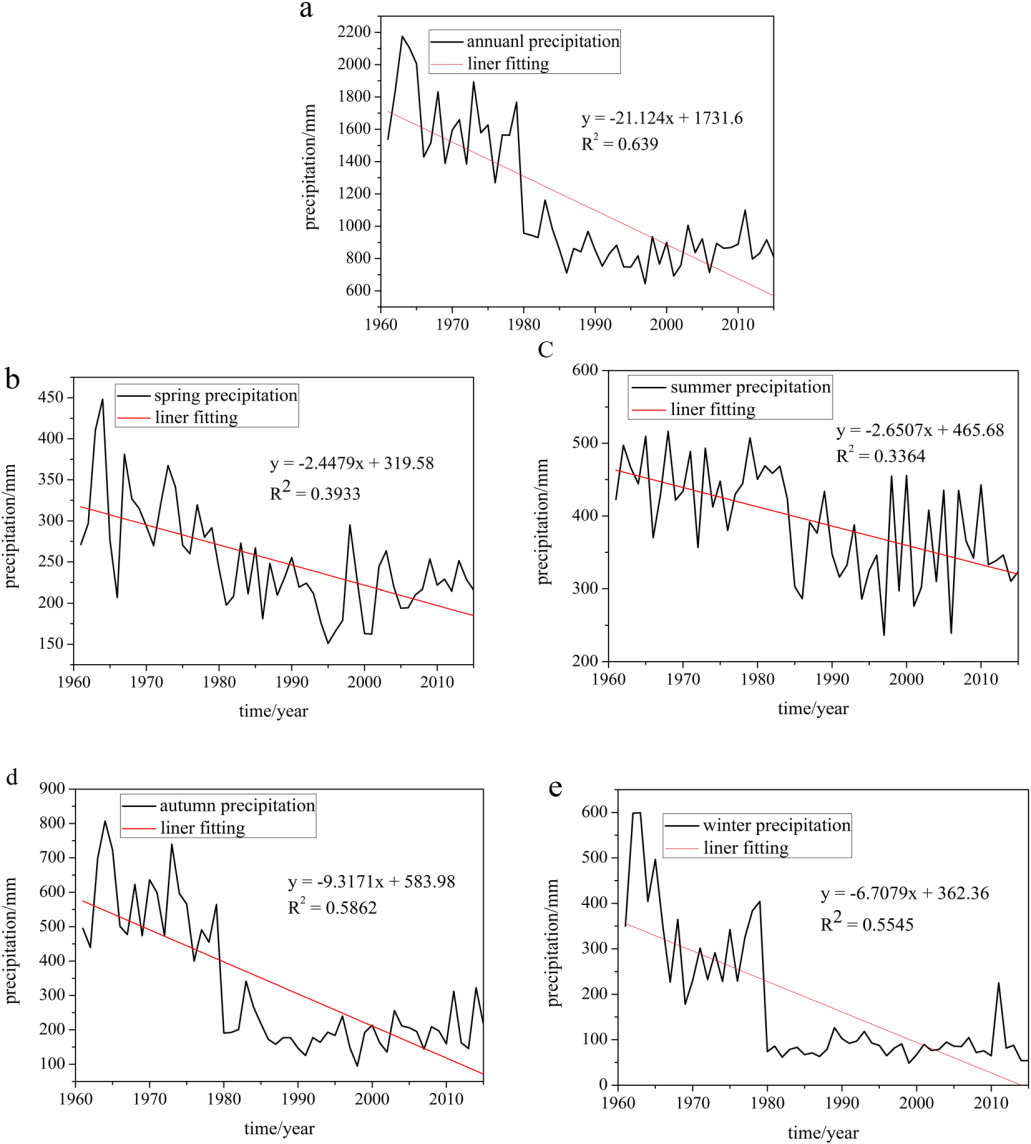
Change trends of the annual and the seasonal precipitation in the Qinba mountain area: (**a**) annual, (**b**) spring, (**c**) summer, (**d**) autumn, and (**e**) winter.

The trends of the annual and the seasonal precipitation were examined using the moving average and linear regression methods. Figure 1 shows the change of annual and seasonal precipitation in the QM. As it can be observed in the figure, the annual precipitation fluctuated between 645 mm and 2175.2 mm (1961–2015), with the minimum and the maximum annual precipitation in 1997 and 1963, respectively. The annual precipitation trend is −21.1 mm/a in the area and the seasonal precipitation trends in the spring, the summer, the autumn, and the winter are −2.45 mm/a, −2.65 mm/a, −9.32 mm/a and −6.71 mm/a, respectively (Fig. 1). The annual precipitation and the seasonal precipitation suggest a downward trend. The trend of annual precipitation and the seasonal precipitation are significant at 95% confidence level.

### Trends in extreme precipitation indices

Table 2 presents the trend and the Mann–Kendall (MK) test of the eight extreme precipitation indices^24^. For the entire study area, SDII, RX1day, RX5day and R95p displayed an increasing trend between 1961 and 2015. However, only the regional trend of the SDII passed the significance test at the 5% significance level. The trend of SDII is 0.024 mm/day/ decade in study area, and SDII had a regionally positive trend at 88.2% of the stations. A decreasing trend was identified in PRCPTOT, in the study area with a value of −1.86 mm/decade. The trend failed the significance test at the 5% significance level, suggesting that the precipitation in the region shows an insignificant trend. R10mm displayed decreasing trend with a value of − 0.006 d/decade, at 94% of the stations. Consecutive wet days (CWD) is an index to measure the extreme of consecutive wet days. The CWD showed a decreasing trend with a value of −0.02 day/decade. Corresponding with the CWD, consecutive dry days (CDD) is an index to measure extreme of consecutive dry days. Based on the CDD, the regional trend was 0.14 d/decade for the study area.

**Table 2:** The results of regional trends and the Mann-Kendall (MK) test for the extreme precipitation indices over the QM during 1961–2015.

Index	Regional Trends		MK Test		Percentage of Stations with Positive Trend	Percentage of Stations with Significant Positive Trend	Percentage of Stations with Negative Trend	Percentage of Stations with Significant Negative Trend
	Units	L	Z	p-Value				
PRCPTOT	mm/decade	−1.8601	−1.7568	0.078	23.5%	0	76.5%	6%
SDII	mm/day/decade	0.0237	2.0399	**0.041**	88.2%	0	11.8%	0
RX1day	mm/decade	0.0609	0.5735	0.569	35.3%	0	64.7%	0
RX5day	mm/decade	0.0246	0.087	0.928	53%	0	47%	0
R95p	mm/decade	0.7046	1.6116	0.107	100%	6%	0	0
R10mm	days/decade	−0.0065	−0.1742	0.865	6%	0	94%	18%
CDD	days/decade	0.1412	1.3575	0.174	76.5%	0	23.5%	0
CWD	days/decade	−0.0168	−1.8584	0.063	23.5%	0	76.5%	0

Notes: L denotes linear trends (decade^−1^), Z is the standardized MK test statistic. Values for statistically significant trends at a 5% significant level are in bold.

A downward trend was identified in PRCPTOT and R10 mm, while the SDII, RX1day, RX5day and R95p had an upward trend. At the same time, a decrease in the CDD and an increase in the CWD, which implied the possibility of increase of the extreme rainfall.

### Abrupt changes of annual and seasonal precipitation

**Figure 2:**
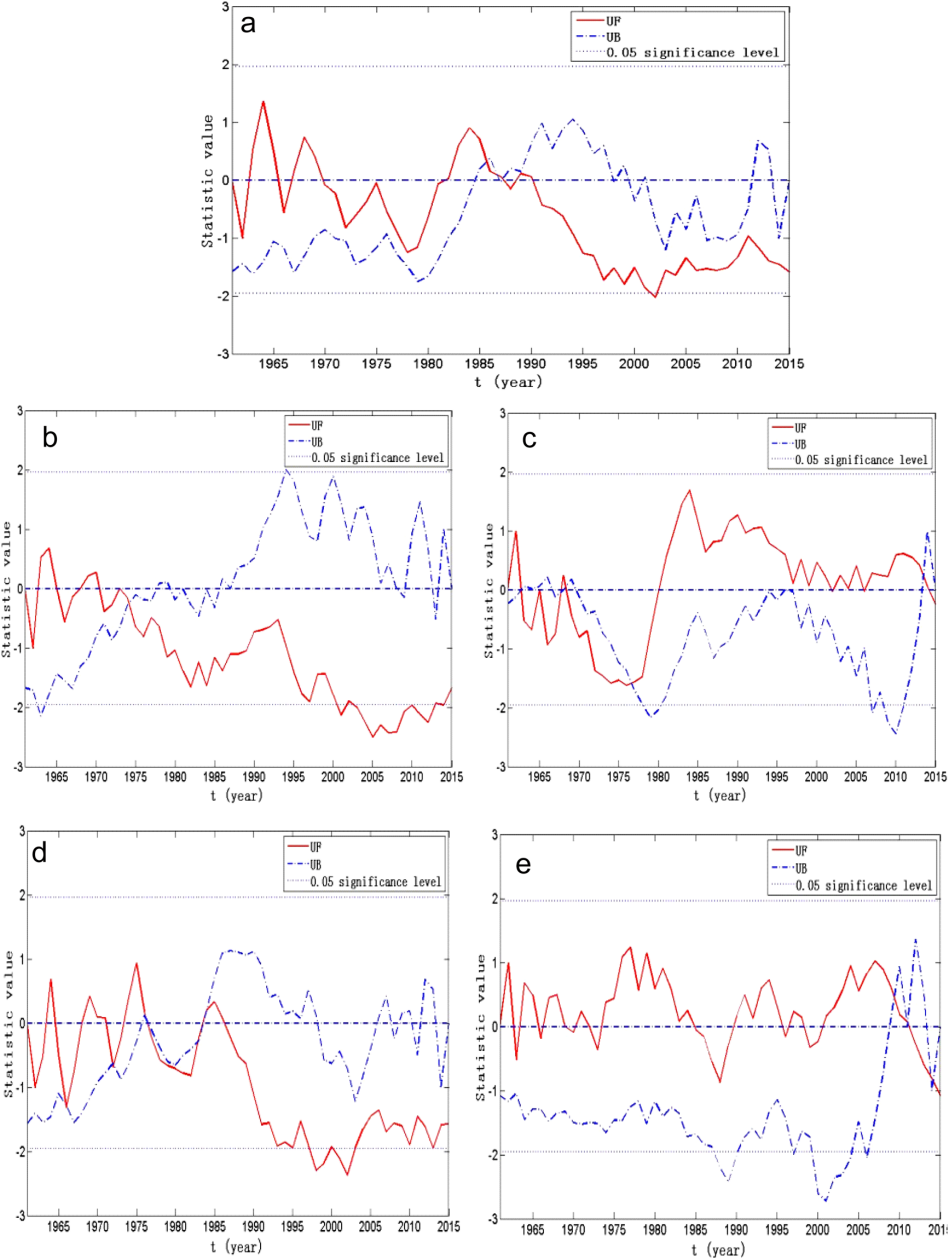
Mann-Kendall statistic curve of the annual and the seasonal precipitation sequence: (**a**) annual, (**b**) spring, (**c**) summer, (**d**) autumn, and (**e**) winter.

Detecting the precipitation change point is an essential factor that can help to provide a better interpretation and more accurate forecast for the hydrological data. The change points of the annual and seasonal precipitation time series are presented in Fig. 2. Abrupt changes were observed in the inter-annual variation of the annual and seasonal precipitation in the study area. Figure 2(a) represents the obvious intersection occurred in 1986, suggesting a sudden decrease in the annual precipitation, after 1986. Figure 2(b) suggests the presence of an obvious intersection in spring precipitation, which occurred in 1973, followed by a significant spring precipitation decrease, between 2000 and 2013 (*P* < 0.05). Figure 2(c) represents obvious intersections in summer precipitation, with the intersections in 1963, 1967, 1970, and 1977. The summer precipitation showed an increasing trend, after 1977. Figure 2(d) displays the obvious intersections in autumn precipitation, with the intersections in 1967, 1972, 1976, 1983, and 1984. The autumn precipitation pattern started to show a decreasing trend after 1984, with a significant decrease from 1998 to 2002 (*P* < 0.05). The obvious intersection in winter precipitation, occurred in 2008, can be detected in Fig. 2(e) followed by a decrease after 2008. According to the above analyses, only certain degree of abrupt changes of the annual and seasonal precipitation can be identified in the study area.

### Precipitation ranks analysis

**Figure 3:**
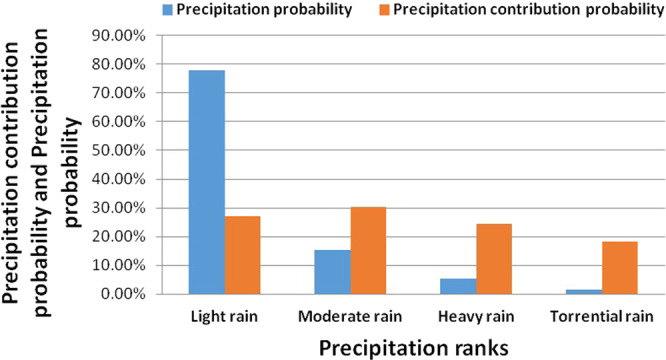
The incidence and contribution rates of different precipitation ranks in the Qinba Mountains.

Figure 3 represents the statistical results of the incidence and contribution rates of different precipitation ranks in the Qinba Mountains. As it can be seen in the figure, the increase of precipitation grade resulted in a decrease in the probability of precipitation. The probability of the light rain, the moderate rain, the heavy rain, and the torrential rain are 77.94%, 15.32%, 5.35%, and 1.39%, respectively. The probability of light rain is dominant in the area. In terms of different levels of precipitation contribution rate, the contribution rates of the light rain, the moderate rain, the heavy rain, and the torrential rain are 26.95%, 30.4%, 24.34%, and 18.31%, respectively.

### Morlet wavelet analysis

**Figure 4:**
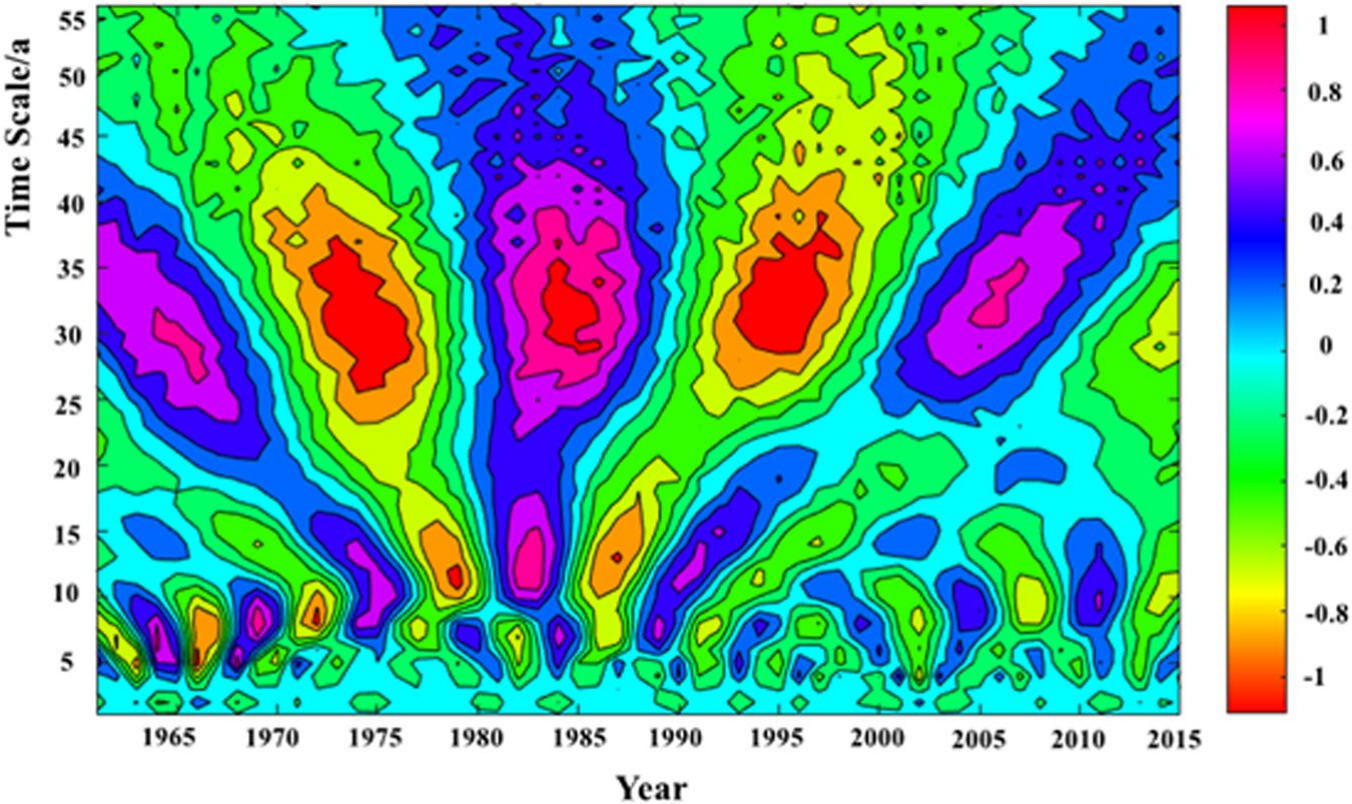
The annual precipitation Morlet wavelet transform in the study area.

The real-data contour map of the annual precipitation Morlet wavelet transform in the Qinba Mountain area, is presented in Fig. 4. Based on the contour map, the annual precipitation has an inter-decadal variation period of 30a~35a on the whole time scale. The central scale is 32a, and the periodic oscillation is very significant. Short periods of 5a~10a are also detected on the inter-annual scale, with the central scale of 8a. The scale period is more obvious during the 1960s to 1970s. In addition, short-period oscillations of 10a~15a are existed on a small scale, before 1990s, and the period remains unclear, after 1990s.

### The probability density function (PDF)

**Figure 5:**
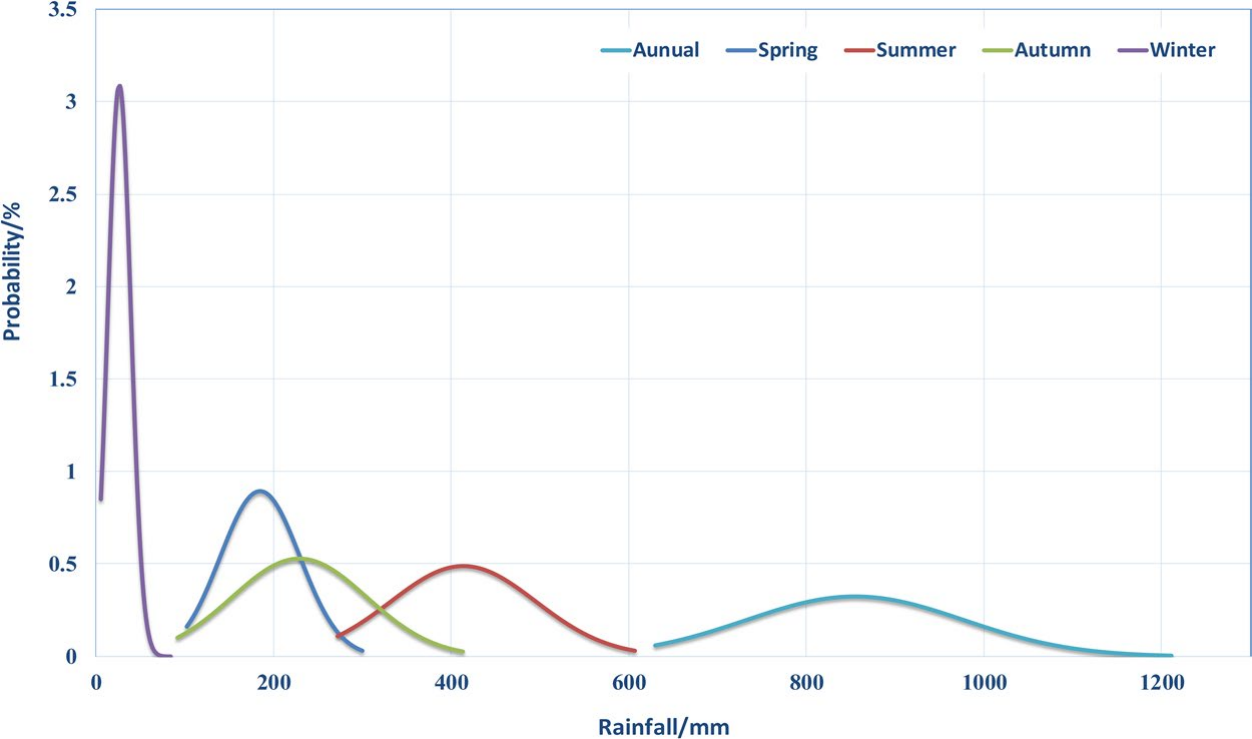
The probability density function (PDF) of the annual and the seasonal precipitation sequences in the Qinba Mountains.

The probability density function (PDF) was used to analyse the annual and seasonal precipitation sequences in the study area. The presented results in Fig. 5 suggest that the precipitation in the Qinba Mountain area accords with the Gaussian normal probability distribution. Furthermore, the PDF curves of the annual precipitation and the seasonal precipitation are bimodal, while, the PDF curve of the winter precipitation is identified as the steepest and the highest peak, and located close to the horizontal and vertical coordinates. This indicates that the winter precipitation distribution is mainly concentrated in the light rain range, and the probability of its occurrence is small.

### Spatial variation

**Figure 6:**
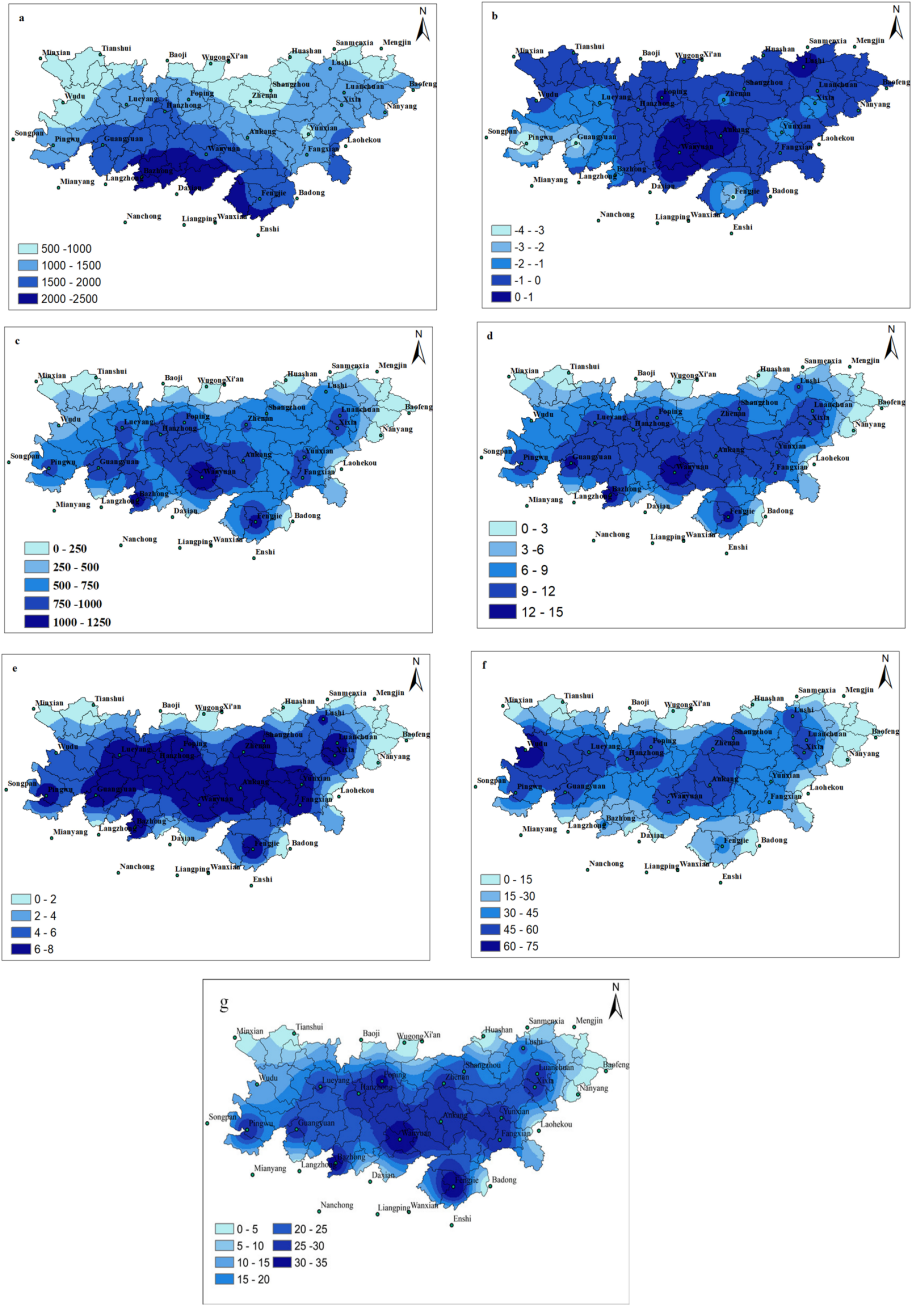
Spatial distribution patterns of the annual precipitation and extreme precipitation indices: (**a**) Annual precipitation, (**b**) Trends in annual precipitation change, (**c**) Annual total wet-day precipitation (PRCPTOT), (**d**) Simple daily intensity index (SDII), (**e**) Consecutive wet day (CWD), (**f**) Consecutive dry days (CDD), (**g**) Number of heavy precipitation days (R10). The maps are generated with Arc Map version 10.1 (http://www.esri.com/software/arcgis).

Figure 6 represents the spatial distribution of precipitation in Qinba Mountain Area. Figure 6(a) shows that the spatial distribution of precipitation between the north and the south is obviously different and it decreases from the south to the north. The areas with the high rate of precipitation are mainly concentrated in the southwest of the study area, and the areas with the lowest rate of precipitation appear in the northwest of the Qinba Mountain Area. Generally speaking, the annual precipitation in most of the region in the study area presents a decreasing pattern (Fig. 6b). The increasing trend of the precipitation are observed in the central and the northeast part of the QM, indicating that flooding may occur in these areas. In contrast the decreasing trend appears in the western, the eastern, and the southern regions of the QM, suggesting the probability of drought occurrence in these areas. Figure 6(c) represents uneven distribution of the PRCPTOT in the study area, with the highest concentration in the southwest. Figure 6(d) shows that the SDII of the central region is fluctuating between 9 and 15, and the largest value of the SDII are observed in Wanyuan, Bazhong, Guangyuan, and Fengjie, fluctuating between 12 and 15. Figure 6(e) shows that the highest rate of the CWD is observed in the central and surrounding areas of the study area, with a value between 6 and 8. The highest rate of the CDD occurs in the central and the western parts of the study area, with more concentration in the western region (Fig. 6(f)). Figure 6(g) suggests the areas with R10 are mainly located in the central and the surrounding areas of the study area. Based on these information, floods are likely to occur in the central part of the study area.

### The relationship between precipitation and altitude, latitude and longitude

**Figure 7:**
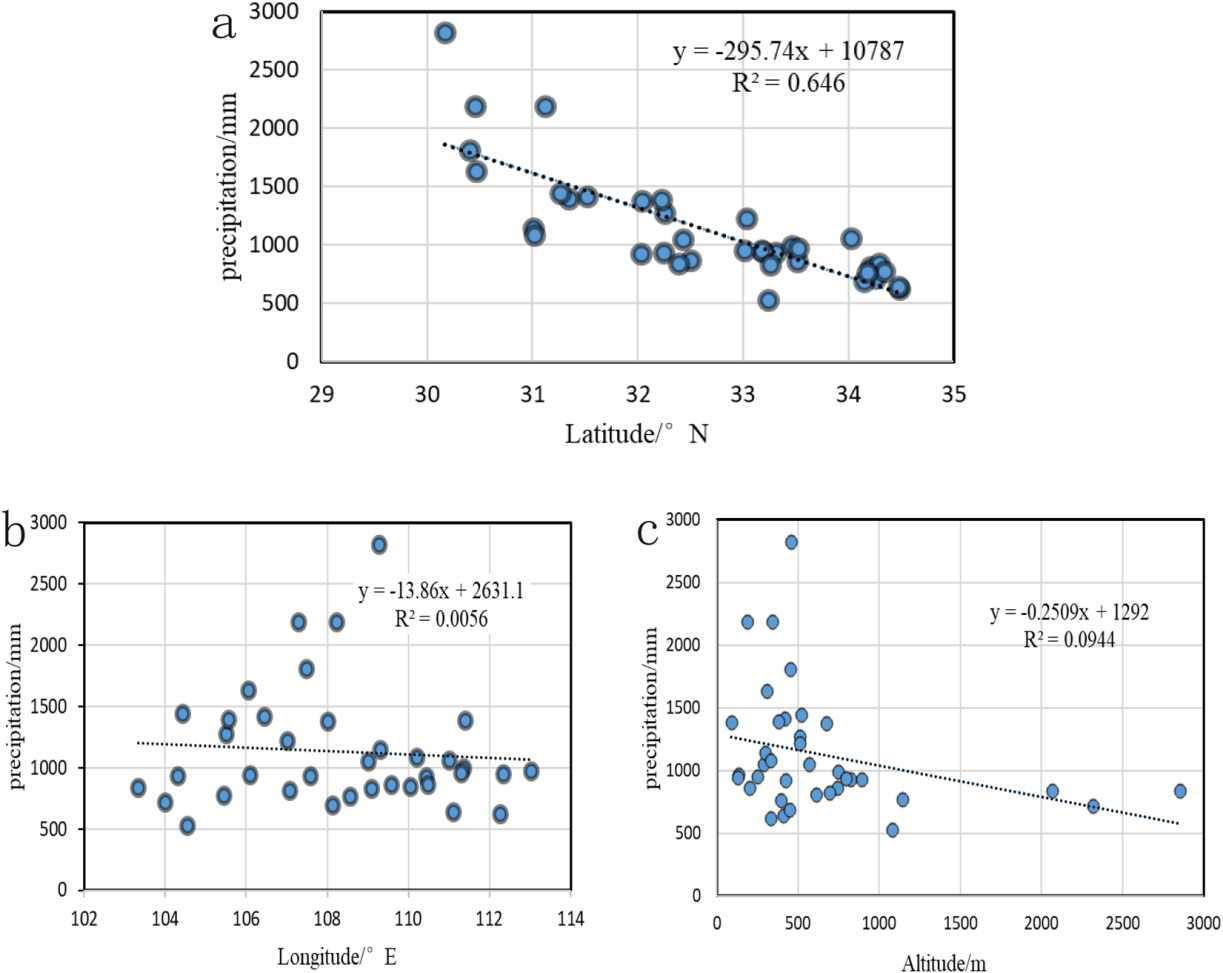
The relationship between the precipitation and the longitude, the latitude, and the altitude.

The spatial distribution of the precipitation in the study area also varies greatly in different areas and the precipitation is correlated to the latitude, the longitude, and the altitude to a certain extent^25^. As it can be observed in Fig. 7 the precipitation decreases with the increase in the longitude, the latitude, and the altitude, and the amount of precipitation decreases significantly with the increase of the latitude. Based on the Table 3, the correlation coefficient R between the precipitation and the latitude is −0.804 and it is highly significant at 99% level. The correlation coefficient R of the precipitation and the longitude is −0.075 and it is very insignificant, with no obvious relationship between the precipitation and the longitude. Furthermore, an insignificant correlation coefficient R is present between the precipitation and the altitude, which is −0.307. Therefore, it can be concluded that the precipitation is mainly related to the latitudinal zonation in the study area.

**Table 3:** The correlation coefficients between the precipitation and the longitude, the latitude, and the altitude.

	precipitation	latitude	longitude	altitude
precipitation	1	−0.804**	−0.075	−0.307

**Denotes Significant at the 0.01 level.

## Discussion

Based on the current literature^26,27^, a downward trend was observed in the national precipitation, after 1980, and the annual precipitation showed a downward trend in the northeast and the northwest of China. R10mm and the CWD all showed a downward trend in the Yangtze River Basin^6,7^, while the CDD showed an upward trend, especially as the SDII, the RX1day, the RX5day, and the R95p also showed an upward trend. The results from our study are basically consistent with the previous results, however, the mutation points of precipitation changes in various regions are different.

The annual precipitation gradually decreases from the south to the north, which is in agreement with the spatial of precipitation, in China^28^. The maximum SDII and positive trends of precipitation are observed in the central and the northeast part of study area. The CWD and the R10 are more in the central part of the study area. Meanwhile, the negative trends appear in the western and the southern regions of the study area, with a higher rate of CDD in the central and the western regions of the study area. On this basis, extreme precipitation is likely to occur in the central region, which may lead to either the regional floods, or the regional droughts.

Atmospheric circulation changes are considered as important mechanisms, which affect the regional heat and water transport^29^. Due to the location of the study area, which is located in the eastern part of the Qinghai-Tibet Plateau and in the central part of the Yangtze River Basin, the precipitation changes are largely affected by the monsoon climate. The monsoon climate change in the Yangtze River Basin can determine the amount of precipitation, to a certain extent. Yang Bo *et al*. (2014) suggested that the extreme precipitation events in the Qinba Mountain area may be affected by the southwest monsoon, which has a great influence on the climate of the surrounding area^30^. El Niño-Southern Oscillation(ENSO)has certain influence on the precipitation changes during the different seasons^31^. At the same time, the influence of topography on the precipitation distribution and extreme precipitation variation is deniable^32^. Indeed, due to the special geographical location of the study area and the type of landforms in the mountainous hills, the types of climate are diverse, and the deriving factors which affect the precipitation change remain unclear. The latter explains the necessity of conducting further study to identify the impact of extreme precipitation on climate change and its future trends.

## Conclusions

The main finding of this study are as follows:

1. In general, the annual precipitation and the seasonal precipitation showed a downward trend, and the annual precipitation decreased after 1986. The annual precipitation suggested an inter-decadal variation period of 30a~ 35a on the whole time scale, with a significant periodic oscillation. The increase in the precipitation rank resulted in decrease of the incidence of precipitation.
2. Only the Regional trend of the SDII passed the significance test at the 5% significance level, among the eight precipitation extreme indices. The SDII, the RX1day, the RX5day, and the R95p displayed an increasing trend, while the PRCPTOT displayed a decreasing trend, during 1961–2015. Analysing the relationship between the precipitation and the longitude, the latitude, and the altitude, we identified that the precipitation is greatly related to latitudinal zonation, in the study area.
3. The uneven spatial distribution of precipitation in the study area suggests a higher rate of the annual precipitation in the central and the southwest regions, in comparison with the other regions. The areas with the CWD and R10 are mainly located in the central and the surrounding areas of the study area, while the areas with CDD are mainly existed in the central and the western parts of the study area.

To conclude, this research studied the temporal and spatial variation characteristics of precipitation in the area, based on the meteorological data. The results from this study can reflect the overall precipitation changes in the region. Due to the limited selection of the meteorological data, the precipitation changes in some areas are not necessarily accurately reflected. The regional runoff changes and the high-precision precipitation data can more accurately reflect the regional floods and the drought conditions, which can be subjected to study, in future.

### Study area

Qinba Mountain is located in the central China, between 102° 54’E ~ 113° 40’E and 30° 50’N ~ 34° 59’N, with a total area of 225,000 km^2^. It sits east of the Qinghai-Tibet Plateau, and the landforms are dominated by the mountains. The Qinba Mountain area is an important ecological barrier in the upper reaches of the Yangtze River. The forests, the grass resources, the regional products, and the mineral resources are extremely rich. The area is not only the main grain-producing land, but also rich in dozens of regional products such as silk, ramie, tea, lacquer. The Qinba Mountain area is also very rich in the underground treasures, and is considered as an important non-ferrous metal and precious metal deposit area, in China.

### Data

**Figure 8:**
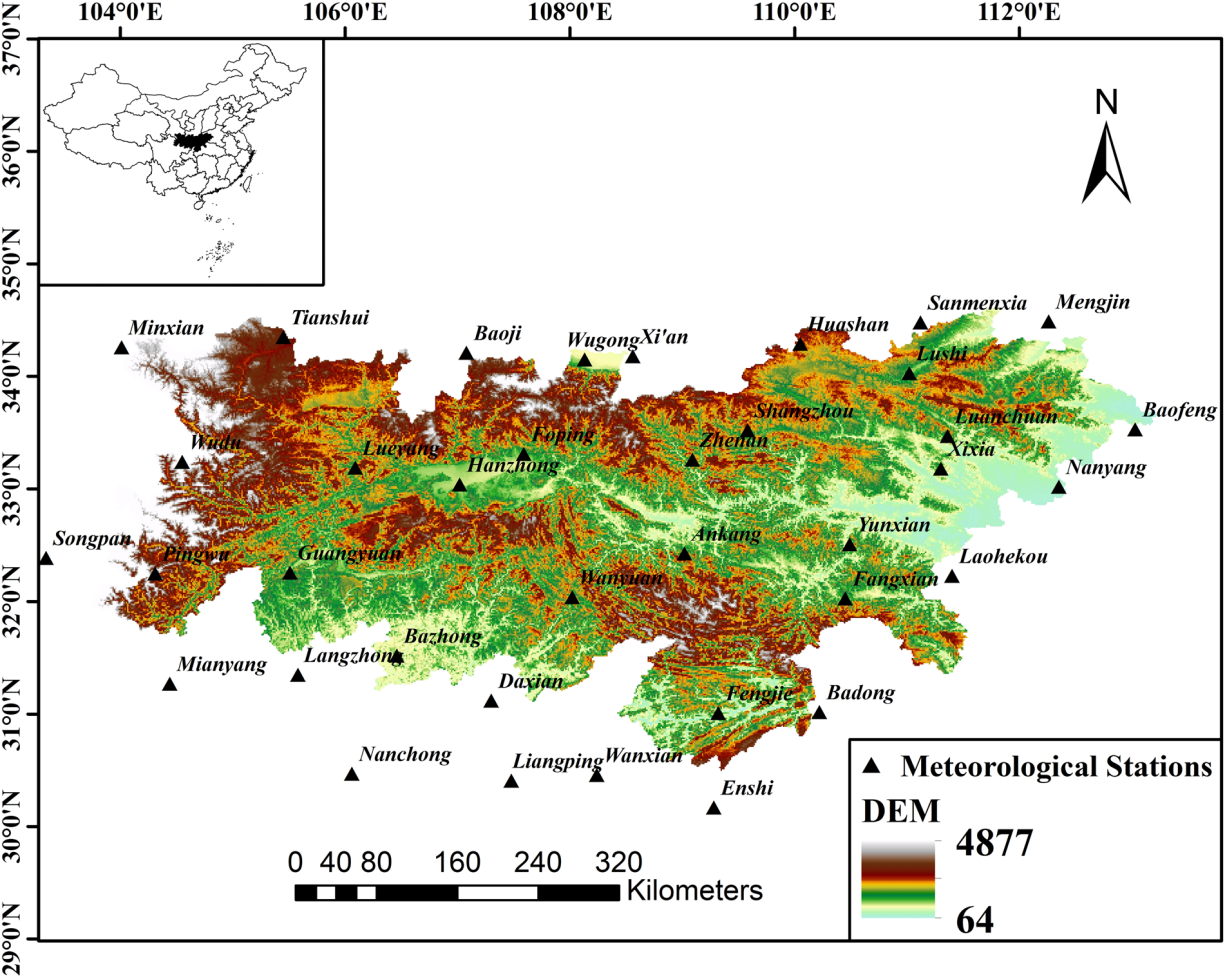
Distribution of weather stations in the region. The maps are generated with Arc Map version 10.1 (http://www.esri.com/software/arcgis).

In this study, the daily precipitation data from 1961 to 2015 were selected from 37 sites (including 17 sites in the study area) with long, uniform, and representative precipitation in Qinba and surrounding areas (Fig. 8). Meteorological data were collected from the China Meteorological Data Sharing Service Network (http://data.cma.cn/), and data quality control was performed by mean of the RClimDex software. Missing data from the Meteorological Stations were interpolated using the Kriging interpolation. Missing data of one day or two days were filled by average values of the neighbouring days. If consecutive days had missing data, the missing values were replaced with the long-term averages of the same days^33,34^.

The meteorological station′s detailed information are presented in Table 4. The selected stations are spread out over the study area, unevenly. The seasons were define as follows: the winter (December, January, and February), the spring (March, April, and May), the summer (June, July, and August), and the autumn (September, October, and November).

**Table 4:** Detailed information of the selected stations.

Station number	Name	Latitude(°N)	Longitude(°E)	Altitude(m)
57245	Ankang	32.43	109.02	290.8
57313	Bazhong	31.52	106.46	417.7
57259	Fangxian	32.03	110.45	426.9
57348	Fengjie	31.01	109.32	299.8
57134	Foping	33.31	107.59	827.2
57206	Guangyuan	32.26	105.51	513.8
57127	Hanzhong	33.04	107.02	509.5
57067	Lushi	34.03	111.02	568.8
57077	Luanchuan	33.47	111.36	750.3
57106	Lueyang	33.19	106.09	794.2
56193	Pingwu	32.25	104.31	893.2
57143	Shangzhou	33.52	109.58	742.2
57237	Wanyuan	32.04	108.02	674
56096	Wudu	33.24	104.55	1079.1
57156	Xixia	33.18	111.3	250.3
57253	Yunxian	32.51	110.49	201.9
57144	Zhenan	33.26	109.09	693.7
57355	Badong	31.02	110.22	334
57181	Baofeng	33.53	113.03	136.4
57016	Baoji	34.21	107.08	612.4
57328	Daxian	31.12	107.3	344.9
57447	Enshi	30.17	109.28	457.1
57046	Huashan	34.29	110.05	2064.9
57306	Langzhong	31.35	105.58	382.6
57265	Laohekou	32.23	111.4	90
57426	Liangping	30.41	107.48	454.5
57071	Mengjin	34.49	112.26	333.3
56196	Mianyang	31.27	104.44	522.7
56093	Minxian	34.26	104.01	2315
57411	Nanchong	30.47	106.06	309.7
57178	Nanyang	33.02	112.35	129.2
57051	Sanmenxia	34.48	111.12	409.9
56182	Songpan	32.39	103.34	2850.7
57006	Tianshui	34.35	105.45	1141.7
57432	Wanxian	30.46	108.24	186.7
57034	Wugong	34.15	108.13	447.8
57036	Xi’an	34.18	108.56	397.5

Consequently, the annual precipitation, the monthly precipitation, the seasonal precipitation, and the eight precipitation extreme indices were discussed in this study. The related concepts of precipitation extreme indices are presented in Table 5^35^.

**Table 5:** The precipitation indices of the study.

Index	Descriptive concept	Index Definitions	Units
PRCPTOT	Annual total wet-day precipitation	Annual total precipitation (PR ≥ 1 mm)	mm
RX1day	Maximum 1-day precipitation	Maximum 1-day precipitation	mm
RX5day	Maximum 5-day precipitation	Maximum consecutive 5-day precipitation	mm
R95p	Very wet day precipitation	Total precipitation (PR>95th percentile of days with PR ≥ 1 mm)	mm
R10mm	Number of heavy precipitation days	Annual count of days when PR ≥ 10mm	days
CDD	Consecutive dry days	Maximum number of consecutive days with PR <1 mm	days
CWD	Consecutive wet days	Maximum number of consecutive days with PR ≥ 1 mm	days
SDII	Simple daily intensity index	Average daily precipitation when PR ≥ 1 mm	Mm/day

## Methods

### Linear regression method

The linear regression method is employed to investigate and analyse the long-term trends of the precipitation, in the time series. The main statistical parameter (i.e. the slope) is used to indicate the temporal change of the studied variable on the spatial scale. This method intuitively reflects the trend of the rainfall time series, and the slope of the linear equation represents the average change rate of the trend (unit: mm/y). The slope can be calculated using the Eq. (1), as follows:

1 $${\rm{Slope}}=\frac{{\rm{n}}\times {\sum }_{{\rm{i}}=1}^{{\rm{n}}}({\rm{i}}\times {{\rm{p}}}_{{\rm{i}}})-{\sum }_{{\rm{i}}=1}^{{\rm{n}}}{\rm{i}}{\sum }_{{\rm{i}}=1}^{{\rm{n}}}{{\rm{p}}}_{{\rm{i}}}}{{\rm{n}}\times {\sum }_{{\rm{i}}=1}^{{\rm{n}}}{{\rm{i}}}^{2}-{({\sum }_{{\rm{i}}=1}^{{\rm{n}}}{\rm{i}})}^{2}}$$

where *slope* represents the estimated linear trend of precipitation during the period of 1961 to 2015. The positive values of the slope indicate the increasing trend, while the negative values of the slope denote the decreasing trend; *i* is the number of years in the time series (i.e. from 1 to 55, in our study); and ${p}_{i}$ is the annual precipitation amount^36^.

### Mann-kendall method

The Mann-Kendall method (M-K) has been widely used to assess trends in hydro-climatic data, Hence, we used this method to test the trends of precipitation in the study area.

The M-K method is a non-parametric method, and the World Meteorological Organization strongly recommends this method for analyses of the hydrological series, because this method does not require any distribution assumptions on the data^37^.

Mann-Kendall trend test is defined as follows:

2 $${\rm{T}}=\mathop{\sum }\limits_{{\rm{i}}=1}^{{\rm{m}}-1}\,\mathop{\sum }\limits_{{\rm{j}}={\rm{i}}+1}^{{\rm{m}}}\,{\rm{sgn}}({{\rm{x}}}_{{\rm{i}}}-{{\rm{x}}}_{{\rm{j}}})$$

Where T is a value, x_i_ and x_j_ are the sequential precipitation data values, m is the Number of time series, and sgn (x_i_-x_j_) is expressed as follows:

3 $${\rm{sgn}}({{\rm{x}}}_{{\rm{i}}}-{{\rm{x}}}_{{\rm{j}}})=\{\begin{array}{llll}1 & if\,{{\rm{x}}}_{{\rm{i}}} &  >  & {{\rm{x}}}_{{\rm{j}}}\\ 0 & if\,{{\rm{x}}}_{{\rm{i}}} & = & {{\rm{x}}}_{{\rm{j}}}\\ -1 & if\,{{\rm{x}}}_{{\rm{i}}} &  <  & {{\rm{x}}}_{{\rm{j}}}\end{array}$$

The variance of the T statistic is expressed as follows:

4 $${\rm{Var}}({\rm{T}})=\frac{{\rm{m}}({\rm{m}}-1)(2{\rm{m}}+5)}{18}$$

And the test statistic Z is expressed as follows:

5 $${\rm{Z}}=\{\begin{array}{lll}\frac{{\rm{T}}-1}{\sqrt{{\rm{Var}}({\rm{T}})}} & if & {\rm{T}} > 0\\ 0 & if & {\rm{T}}=0\\ \frac{{\rm{T}}+1}{\sqrt{{\rm{Var}}({\rm{T}})}} & if & {\rm{T}} < 0\end{array}$$

The Z value is used to calculate the statistically significant trend. A positive value of Z represents an upward trend, while a negative value of Z suggests a downward trend. Z_α_/2 is the critical value of the standard normal distribution. If $\,|{\rm{Z}}|\ge {{\rm{Z}}}_{{\rm{\alpha }}/2}$, the trend is significant, while $|{\rm{Z}}| < {{\rm{Z}}}_{{\rm{\alpha }}/2}$, represents an insignificant trend.

In this study, the M-K test is used to detect whether a trend in the rainfall time series is statistically significant at a 95% confidence level. Mann-Kendall test can be further used to test sequence mutation, as follows:

6 $${{\rm{UF}}}_{{\rm{K}}}=\frac{|{{\rm{S}}}_{{\rm{K}}}-{\rm{E}}({{\rm{S}}}_{{\rm{K}}})|}{\sqrt{{\rm{Var}}({{\rm{S}}}_{{\rm{K}}})}}\,(k=1,\,2,\,\ldots ,n)$$

Where $${\rm{E}}({{\rm{S}}}_{{\rm{k}}})={\rm{k}}({\rm{k}}-1)/4$$; $${\rm{Var}}({{\rm{S}}}_{{\rm{k}}})={\rm{k}}({\rm{k}}-1)(2{\rm{k}}+5)/72$$

UF_K_ is the standard normal distribution, given level of significance *α*, if UF_K_ > U_α/2_, which indicated the sequence trend. The time series X is arranged in reverse order, and subsequently calculated according to the Eq. 6.

7 $$\{\begin{array}{c}{{\rm{UB}}}_{{\rm{K}}}=-{{\rm{UF}}}_{{\rm{K}}}\\ k=n+1-k\end{array}\,(k=1,\,2,\ldots ,n)$$

The trend of sequence X can be further analysed by UF_K_ and UB_K_ presented in Eq. 7. If UF_K_ >0, the sequence is increasing, while, if UF_K_ <0, the sequence is decreasing. The mutation time can also be determined and the mutation region is pointed out. In this regard, exceeding the critical line suggest that the trend of rise or fall is significant. If the 2 curves of UF_K_ and UB_K_ intersect, and the intersection point is between the critical straight lines, then the starting point of the mutation is the corresponding moment of the intersection point^38^.

### Precipitation ranks analysis

According to the daily precipitation in the national standard, the precipitation grade is divided into four categories including the light rain (when the daily precipitation is 0.1–9.9 mm), the moderate rain (when the daily precipitation is 10–24.9 mm), the heavy rain (refers to the daily precipitation of 25–50 mm), the torrential rain (refers to the daily precipitation ≥50 mm).

In order to comprehensively evaluate the variation characteristics of precipitation in the study area, this research introduces two indicators including the precipitation occurrence rate and the precipitation contribution rate. The precipitation occurrence rate refers to the proportion of occurrences of various precipitation events, in a certain classification. The precipitation contribution rate is defined as the ratio of precipitation to the total precipitation, in a certain classification.

### The wavelet analysis method and the probability density function

The wavelet analysis method has multiple modes and in this study the Morlet transform analysis has been used to analyse the data^39^.

The probability density function (PDF) is a function to describe the probability that the output value of a continuous random variable is near a certain point^40^. In this research, the PDF is adopted to analyse the probability distribution of rainfall in the Qinba Mountain area.

### Spatial interpolation

During recent years, various spatial interpolation methods have been used to investigate the spatial distribution of the precipitation and the meteorological variables. The spatial interpolation was performed using the inverse distance weighted (IDW) interpolation^41^.The selected method for the spatial interpolation and the spatial resolution of the meteorological stations could influence the spatial pattern of precipitation. Based on our finding, the inverse distance weighting (IDW) method provided the lowest mean error, among the three common interpolation methods (i.e. the spline, the ordinary kriging, and the inverse distance weighting), thus the IDW form ArcGIS was applied to describe the spatial variability of precipitation and meteorological variables in the present study^42^.
