# Author Correction: Analysis of Annual and Seasonal Precipitation Variation in the Qinba Mountain area, China

**DOI:** 10.1038/s41598-021-03607-y

**Published:** 2021-12-16

**Authors:** Yannan Zhang, Chuan Liang

**Affiliations:** College of Water Resource and Hydropower, Sichuan University, 610065 Chengdu, China

Correction to: *Scientific Reports* https://doi.org/10.1038/s41598-020-57743-y, published online 22 January 2020

The original version of this Article contained an error in the Acknowledgments section.

“This work was supported by the National Natural Science Foundation of China (Grant No. 61006403).”

now reads:

“This work was supported by the National Natural Science Foundation of China (Grant No. 41271045).”

The original Article has been corrected.

